# The Nitrogen Stress-Repressed sRNA NsrR1 Regulates Expression of *all1871*, a Gene Required for Diazotrophic Growth in *Nostoc* sp. PCC 7120

**DOI:** 10.3390/life10050054

**Published:** 2020-04-29

**Authors:** Isidro Álvarez-Escribano, Manuel Brenes-Álvarez, Elvira Olmedo-Verd, Agustín Vioque, Alicia M. Muro-Pastor

**Affiliations:** Instituto de Bioquímica Vegetal y Fotosíntesis, Consejo Superior de Investigaciones Científicas and Universidad de Sevilla, 41092 Sevilla, Spain; isidroae9@hotmail.com (I.Á.-E.); mabreal92@gmail.com (M.B.-Á.); eolmedoverd@hotmail.com (E.O.-V.); vioque@us.es (A.V.)

**Keywords:** regulatory RNA, cyanobacteria, post-transcriptional regulation, heterocyst

## Abstract

Small regulatory RNAs (sRNAs) are post-transcriptional regulators of bacterial gene expression. In cyanobacteria, the responses to nitrogen availability, that are mostly controlled at the transcriptional level by NtcA, involve also at least two small RNAs, namely NsiR4 (nitrogen stress-induced RNA 4) and NsrR1 (nitrogen stress-repressed RNA 1). Prediction of possible mRNA targets regulated by NsrR1 in *Nostoc* sp. PCC 7120 allowed, in addition to previously described *nblA*, the identification of *all1871*, a nitrogen-regulated gene encoding a protein of unknown function that we describe here as required for growth at the expense of atmospheric nitrogen (N_2_). We show that transcription of *all1871* is induced upon nitrogen step-down independently of NtcA. All1871 accumulation is repressed by NsrR1 and its expression is stronger in heterocysts, specialized cells devoted to N_2_ fixation. We demonstrate specific interaction between NsrR1 and the 5′ untranslated region (UTR) of the *all1871* mRNA, that leads to decreased expression of *all1871*. Because transcription of NsrR1 is partially repressed by NtcA, post-transcriptional regulation by NsrR1 would constitute an indirect way of NtcA-mediated regulation of *all1871*.

## 1. Introduction

Small non-coding RNAs (sRNAs) are relevant players in regulatory circuits affecting essentially every aspect of bacterial physiology. These types of molecules are usually post-transcriptional regulators fine-tuning the responses to different environmental conditions [1]. In fact, the interplay between the regulation exerted by transcription factors and that exerted by small RNAs produces complex regulatory circuits in the form of feed-forward loops (coherent or incoherent) involving a transcription factor, an sRNA and their regulated target(s) [2,3].

Global nitrogen regulation is controlled in cyanobacteria by NtcA, a protein that belongs to the CRP/FNR family of transcriptional regulators [4]. Direct binding of NtcA to the corresponding promoters accounts for regulation of many nitrogen-regulated genes [5,6,7]. However, the mechanisms involved in expression of some nitrogen-regulated genes whose promoters do not contain NtcA binding sites might involve the participation of other NtcA-regulated factor(s). NtcA has been shown to regulate expression of several sRNAs, some of them with a wide distribution among phylogenetically distant cyanobacteria [5,8,9]. Among these sRNAs, NsiR4 (nitrogen-stress inducible RNA 4) is involved in nitrogen assimilation control via regulation of IF7, the inactivating factor of the key enzyme glutamine synthetase [8]. NsrR1 (nitrogen-stress repressed RNA 1) modulates translation of NblA [10], a protein required for the degradation of phycobilisomes that provide amino acids as a source of nitrogen under nitrogen deficiency [11]. Transcription of both *gifA* (encoding IF7) and *nblA* is directly regulated by NtcA [5,12], therefore the post-transcriptional regulation exerted on these transcripts by NsiR4 and NsrR1, respectively, constitutes a second level of NtcA-mediated, indirect regulation. 

In the absence of combined nitrogen, filamentous cyanobacteria such as model strain *Nostoc* sp. PCC 7120 differentiate heterocysts, specialized cells devoted to fixation of atmospheric nitrogen [13,14]. Differentiation of functional heterocysts is ultimately under control of NtcA, but also requires HetR, a regulator specifically involved in cellular differentiation. The nitrogen-regulated, HetR-dependent transcriptome includes transcripts for genes involved in specific aspects of heterocyst physiology, such as the sequential deposition of specialized envelopes or the fixation of nitrogen by the enzyme nitrogenase. The HetR-dependent transcriptome also includes non-coding transcripts, both antisense and small RNAs, that would participate in the metabolic reprogramming that takes place in heterocysts [15,16], again pointing to the relevance of post-transcriptional regulation on cyanobacterial physiology. 

In this work, we identify *all1871* as a gene required for heterocyst function and describe its regulation by NsrR1. Expression of *all1871* is induced upon nitrogen step down, but its induction does not require NtcA or HetR. We verify that NsrR1 regulates accumulation of All1871 at the post-transcriptional level by its interaction with the 5′-UTR of *all1871*.

## 2. Materials and Methods

### 2.1. Strains and Growth Conditions 

Cultures of wild-type and the different mutant derivatives of *Nostoc* sp. PCC 7120 (Table S1) were bubbled with an air/CO_2_ mixture (1% *v*/*v*) and grown photoautotrophically at 30 °C in BG11 medium [17] containing ferric citrate instead of ammonium ferric citrate, lacking NaNO_3_ and containing 6 mM NH_4_Cl, 10 mM NaHCO_3_, and 12 mM *N*-tris(hydroxymethyl)methyl-2-aminoethanesulfonic acid-NaOH (TES) buffer (pH 7.5). Nitrogen deficiency was induced by removal of combined nitrogen. Occasionally, 17.6 mM NaNO_3_ was used as nitrogen source. Solid media were solidified with 1% Difco Agar. Mutant strains were grown in the presence of appropriate antibiotics at the following concentrations: streptomycin (Sm) and spectinomycin (Sp), 2–3 µg/mL each (liquid medium) or 5 µg/mL each (solid medium), neomycin (Nm), 5 µg/mL (liquid medium) or 25 µg/mL (solid medium). *Escherichia coli* strains (Table S1) were grown in Luria-Bertani (LB) medium, supplemented with appropriate antibiotics.

### 2.2. Reporter Assays for In Vivo Verification of Targets 

For the experimental target verification in *E. coli*, we used a previously described reporter system [18] and the superfolder green fluorescent protein (sfGFP) plasmid pXG10-SF [19]. The 5′-UTR of *all1871*, from the transcriptional start site (TSS) at position −137 with respect to the initiation codon (coordinate 2234072) to 60 nucleotides within the coding region, containing the predicted NsrR1 interaction sequence, was amplified from genomic DNA using oligonucleotides 247 and 248 (see Table S2 for oligonucleotide sequences and description). The information about the TSS was taken from [5]. The corresponding polymerase chain reaction (PCR) product was digested with NsiI and XbaI and cloned into the vector pXG10-SF digested with NsiI and NheI, resulting in plasmid pIAE9, bearing a translational fusion of a truncated All1871 protein with sfGFP (see Table S3 for plasmid descriptions). For NsrR1 expression in *E. coli*, plasmid pAVN1 [10] was used (Table S3).

Mutation U51G (Mut-51) was introduced in NsrR1 by overlapping PCR using primer pairs 197 and 296, and 198 and 295, and cloned as described for the wild type version [10] generating plasmid pIAE20 (Table S3). The compensatory mutation in the 5′-UTR of *all1871* (Comp-51) was generated in the same way with primer pairs 247 and 304, and 303 and 248, and cloned as described above for the wild-type version resulting in plasmid pIAE22 (Table S3). The sequences of inserts in plasmids containing NsrR1 and *all1871-*sf*gfp* fusions are shown in Tables S4 and S5, respectively.

For testing various combinations of both plasmids, these were introduced into *E. coli* DH5α. Plasmid pJV300 [20] was used as a control expressing an unrelated RNA. Plasmid pXG0 [18] was used as control for background fluorescence. Fluorescence measurements were done with a microplate reader (Varioskan) using liquid cultures from eight individual colonies bearing each combination of plasmids, and normalized to the OD_600_ of each culture as described previously [21]. Fluorescence was also visualized in *E. coli* cells plated on solid LB medium by excitation with a 302-nm wavelength lamp. 

### 2.3. RNA Isolation, Northern Blot and Primer Extension Analysis 

RNA samples were isolated from cells collected at different times after removing combined nitrogen (ammonium) from the media. Alternatively, cells were grown in media lacking combined nitrogen and RNA was isolated from cells collected at different times after the addition of 10 mM NH_4_Cl and 20 mM TES buffer. Total RNA was isolated using hot phenol as described [22] with modifications [9]. Northern blot hybridization was performed as previously described [23,24]. Strand-specific ^32^P-labelled probes for Northern blot were prepared with Taq DNA polymerase using a PCR fragment as template in a reaction with [α-^32^P]dCTP and one single oligonucleotide as primer (corresponding to the complementary strand of the sRNA or mRNA to be detected). Hybridization to *rnpB* [25] was used as loading and transfer control. Hybridization signals were quantified on a Cyclone Storage Phosphor System with Optiquant software (PerkinElmer). Primer extension analysis of 5′ ends of *all1871* was carried out as previously described [23] using 5 µg of total RNA and oligonucleotide 161 labeled with [γ-^32^P]ATP.

### 2.4. In Vitro Synthesis and Labelling of RNA 

The DNA templates for the in vitro transcription of NsrR1 and *all1871* 5′-UTR RNA were generated by PCR with a forward primer that includes a T7 promoter sequence and three extra Gs upstream the 5′-end of the coded RNA, and a reverse primer corresponding to the 3′ end of the RNA (see Tables S2 and S6). The *all1871* 5′-UTR fragment extends from the TSS at position −137 to 60 nucleotides downstream the translational start. RNA transcripts were generated with the MEGAscript High-Yield Transcription Kit (AM1333, Ambion). After transcription, RNAs were treated with DNase I and purified by phenol and chloroform extraction, ethanol-precipitated at –20 °C, and washed with 70% ethanol. In vitro transcribed RNAs were 5′-labelled and purified as described [10].

### 2.5. In Vitro Structure Probing and Footprinting

We mixed 0.1 pmol (about 50,000 cpm) of labeled NsrR1 RNA in 7 µL with 2 pmol of unlabelled *all1871* 5′-UTR RNA, denatured for 1 min at 95 °C and chilled on ice for 5 min, followed by the addition of 1 µL of 1 mg/mL yeast RNA (Ambion AM7118) and 1 µL of 10× structure buffer (Ambion). The samples were incubated further for 15 min at 37 °C. Treatment with RNase T1, RNase A or lead(II) acetate were performed as described [10].

An alkaline ladder was obtained by incubating 0.2 pmol of 5′-labelled RNA at 95 °C for 3 min in 7.5 µL of alkaline hydrolysis buffer (Ambion) containing 1.5 µg of yeast RNA (Ambion AM7118). Reactions were stopped by the addition of 15 µL of denaturing formamide loading buffer.

RNase T1 G ladders were obtained by incubating 0.1 pmol of 5′-labelled RNA and 1 µL of 1 mg/mL yeast RNA (Ambion AM7118) in 9 µL sequencing buffer (Ambion) for 10 min at 50 °C, followed by the addition of 1 µL of 0.1 U/mL RNase T1 (Ambion AM2283) and incubation at room temperature for 15 min. Reactions were stopped by the addition of 20 µL of Inactivation/Precipitation buffer (Ambion) and incubation at −20 °C for 15 min. The precipitate was washed with 70% ethanol and resuspended in 3–7 µL of denaturing formamide loading buffer.

All samples were run on 10% polyacrylamide, 7 M urea gels and bands visualized with a Cyclone Storage Phosphor System (PerkinElmer).

### 2.6. Expression and Purification of Protein All1871 and Western Blot 

To produce His-tagged All1871 protein, the *all1871* gene was amplified using *Nostoc* DNA as template and primers 335 and 336, and the PCR product was cloned in vector pET-28a (+) (Novagen) using NcoI and XhoI, producing plasmid pIAE30. Plasmid pIAE30, containing downstream a T7 polymerase-dependent promoter the *all1871* gene fused at the 3′ end to a sequence encoding a His_6_-tag, was transferred by electroporation to *E. coli* BL21-(DE3)-RIL, in which the gene encoding T7 RNA polymerase is under the control of an isopropyl-β-D-1-thiogalactopyranoside (IPTG)-regulated promoter. A 25-mL pre-inoculum of this strain was grown overnight in LB medium supplemented with chloramphenicol and kanamycin and used to inoculate 275 mL of the same medium. The culture was incubated at 37 °C until OD_600_ = 0.6. Recombinant All1871 expression was induced by the addition of 1 mM IPTG. After 4 h at 37 °C, cells were collected and resuspended in 20 mM sodium phosphate buffer (pH 7.2) containing 6 M urea, 500 mM NaCl, 5 mM imidazole and 1 mM phenylmethylsulfonyl fluoride (6 mL/g of cells). Cells were broken by sonication and after centrifugation at 15,000× *g* (15 min, 4 °C), and the His_6_-All1871 protein was purified from the supernatant by chromatography through a 1-mL HisTrap HP column (GE Healthcare), using an imidazole gradient in the same buffer to elute the retained proteins. An additional purification step was carried out by size-exclusion chromatography in a HiLoad 16/60 Superdex 75 column (Pharmacia) using 20 mM sodium phosphate buffer (pH 7.2) containing 2 M urea and 150 mM NaCl. A total amount of 3.5 mg of purified protein was used in seven subcutaneous injections of a rabbit to produce antibodies in the Animal Production and Experimentation Center, Universidad de Sevilla (Seville, Spain). Antiserum was recovered several times up to five months after the first injection and stored at −80 °C until used. Antibodies specific for All1871 were purified from the serum by affinity chromatography on immobilized His-tagged All1871 using the AminoLink^®^ Plus Immobilization Kit (ThermoFisher, Waltham, MA, USA) following the manufacturer’s instructions. 

For Western blot analysis, *E. coli* cells were resuspended in sodium dodecyl sulphate-polyacrylamide gel electrophoresis (SDS-PAGE) loading buffer and the proteins fractionated on 15% SDS-PAGE. Antibodies against All1871 (see above), GFP (Roche) and *E. coli* GroEL (SIGMA-ALDRICH) were used. The ECL Plus immunoblotting system (GE Healthcare) was used to detect the different antibodies using anti-rabbit (SIGMA-ALDRICH) or anti-mouse (Bio-Rad) horse radish peroxidase conjugated secondary antibodies.

For Western blot analysis of *Nostoc* proteins, soluble fractions of cell-free extracts were used. Cells from 25 mL of culture were resuspended in 500 µL of Tris-HCl buffer (pH 8) containing 2 mM β-mercaptoethanol and protease inhibitor cocktail (cOmplete^TM^ ultra tablets, EDTA-free, Roche) in an Eppendorf 1.5 mL tube. A volume of approximately 75 µL of glass beads (0.25–0.3 mm diameter) was added and the suspension subjected to seven cycles of 1 min vortex followed by 1 min on ice. The resulting extract was centrifuged 3 min at 3000× *g* at 4 °C and the supernatant further centrifuged for 30 min at 16,000× *g* at 4 °C. The supernatant of the last centrifugation constitutes the soluble fraction.

### 2.7. Generation and Complementation of all1871 Mutant Strain 

To generate a strain lacking *all1871*, two overlapping fragments were amplified by PCR using as template genomic DNA with oligonucleotides 162 and 163 and oligonucleotides 164 and 165, respectively (Table S2). The resulting products were then used as templates for a third PCR with oligonucleotides 162 and 165 resulting in deletion of sequences corresponding to *all1871* and the generation of a unique XhoI site between the two amplified fragments. The fragment was cloned into pSpark (Canvax Biotech), rendering pSAM318 and its sequence was verified (Eurofins Genomics). After digestion with BamHI at the sites provided by oligonucleotides 162 and 165, the fragment was cloned into BamHI-digested *sacB*-containing Sm^R^Sp^R^ vector pCSRO [26], rendering pSAM324. A Nm^R^ gene was excised from pRL278 [27] as a SalI-XhoI fragment and cloned into the XhoI site in pSAM324, rendering pSAM326, which was transferred to *Nostoc* sp. strain PCC 7120 by conjugation [28], with selection for resistance to Nm. Cultures of the exconjugants obtained were used to select for clones resistant to 5% sucrose [29], and individual sucrose resistant colonies were checked by PCR using flanking oligonucleotides 162 and 180. Clones bearing the *all1871* region interrupted by the Nm^R^ gene were named *all1871*::Nm. 

Plasmid pIAE65 was constructed to express *all1871* from the *trc* promoter. A PCR fragment was amplified using oligonucleotides 247 and 735, digested with NsiI and XhoI and cloned into NsiI and XhoI-digested pMBA37 [15]. pIAE65 was transferred to strain *all1871*::Nm by conjugation with selection of Sm^R^ Sp^R^ cells.

### 2.8. Construction of a Strain Bearing a Translational Fusion all1871-gfpmut2 

In order to analyze spatial expression of *all1871* along *Nostoc* filaments, plasmid pELV75 (Table S3) was constructed. A fragment containing the sequence from position −200 with respect to the TSS plus the entire *all1871* gene was amplified using oligonucleotides 451 and 596. The fragment was digested with ClaI and EcoRV and cloned in pCSAM147 [30], in frame with the *gpfmut2* gene, rendering pELV73. The EcoRI fragment from pELV73 containing the *all1871-gfpmut2* translational fusion was cloned into pCSV3 [31] producing pELV75, that was transferred to *Nostoc* by conjugation with selection for Sm^R^Sp^R^ cells.

### 2.9. Microscopy 

Fluorescence of *Nostoc* sp. PCC 7120 filaments carrying plasmid pELV75 growing on top of solidified nitrogen-free medium, was analyzed by confocal microscopy and quantified as described [32] using a Leica HCX PLAN-APO 63× 1.4 NA oil immersion objective attached to a Leica TCS SP2 laser-scanning confocal microscope. Samples were excited at 488 nm by an argon ion laser and the fluorescent emission was monitored by collection across windows of 500–538 nm (GFP) and 630–700 nm (cyanobacterial autofluorescence). Filaments were stained with Alcian blue and visualized at the microscope as described [33].

## 3. Results

### 3.1. NsrR1 Interacts with all1871 mRNA 5′-UTR

NsrR1 (nitrogen stress-repressed RNA 1) is a small RNA previously described to post-transcriptionally regulate expression of *nblA* [10]. A prediction of mRNAs possibly regulated by NsrR1 also identified the 5′-UTR of gene *all1871* as likely interacting with NsrR1 [10]. *all1871* would encode a conserved protein of unknown function. The high score of the prediction (*p*-value = 6.11 × 10^−9^), together with the conservation of predicted interactions between NsrR1 homologs and *all1871* homologs in most cyanobacteria encoding NsrR1 (Figure S1), prompted us to further analyze a possible post-transcriptional regulation of *all1871* by NsrR1.

**Figure 1:**
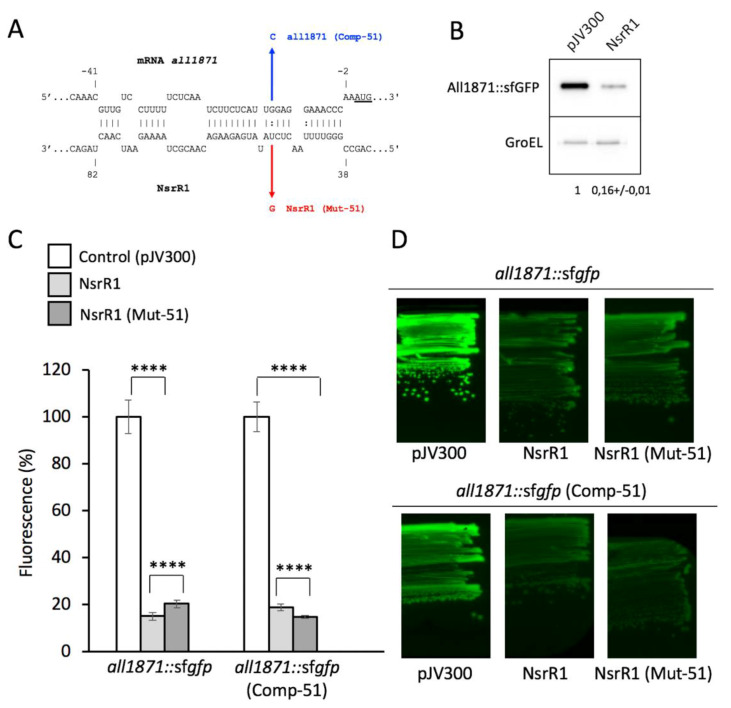
Verification of NsrR1 interaction with the 5′-UTR of *all1871* using an in vivo reporter system. (**A**) Predicted interaction between NsrR1 and the 5′-UTR of *all1871* according to IntaRNA [34]. *all1871* nucleotides are numbered with respect to the start of the coding sequence (AUG start codon is underlined). The mutation introduced in NsrR1 at position 51 (U to G, Mut-51) and the corresponding compensatory mutation in *all1871* 5′-UTR position −13 (G to C, Comp-51) are indicated in red and blue, respectively. (**B**) Accumulation of GFP protein in *E. coli* DH5α cells bearing an *all1871*::sf*gfp* fusion combined with plasmid pJV300 (encoding a control RNA) or with a plasmid encoding NsrR1. Western blots were carried out using antibodies against GFP or GroEL. Numbers at the bottom of the image indicate relative GFP levels with respect to control after normalization with GroEL (average of two experiments). (**C**) Fluorescence measurements of *E. coli* DH5α cultures bearing combinations of plasmids expressing different versions of NsrR1 (wild type or Mut-51) and *all1871*::sf*gfp* fusions (wild type or Comp-51). Plasmid pJV300 (encoding a control RNA) was used as control. The data are presented as the mean +/- standard deviation of cultures from eight independent colonies after subtraction of fluorescence in cells bearing pXG0. Fluorescence is normalized to the OD_600_ of each culture. *T*-test *p*-value < 0.0001****. (**D**) Fluorescence intensities of *E coli* cells bearing *all1871*::sf*gfp* fusions (wild type or Comp-51) combined with different versions of NsrR1 (wild type or Mut-51) or with the control plasmid pJV300. Strains growing on LB agar plates were photographed under ultraviolet (UV) light.

Figure 1 shows the predicted interaction between NsrR1 from *Nostoc* sp. PCC 7120 and the 5′-UTR of *all1871*, that extends from position −40 to −3, overlaps the translation initiation region and therefore is expected to affect translation of the mRNA (Figure 1A). To verify the interaction between NsrR1 and the mRNA of *all1871*, we used a heterologous reporter assay in *E. coli* [18], in which the 5′-UTR of *all1871*, from the TSS at position −137 with respect to the initiation codon (Mitschke et al., 2011), plus 60 nucleotides of the coding sequence of *all1871* were fused to the sf*gfp* gene and co-expressed in *E. coli* with NsrR1 or with a control RNA. Accumulation of the GFP protein was measured in *E. coli* cells bearing different combinations of plasmids. The translation initiation region of *all1871* was functional in *E. coli* resulting in significant GFP protein accumulation and GFP fluorescence (Figure 1B–D). The GFP fluorescence of cells bearing the *all1871::*sf*gfp* fusion (and the amount of GFP protein) decreased to less than 20% of the control when NsrR1 was co-expressed, indicating a direct interaction between NsrR1 and the 5′-UTR of *all1871*, which affects translation (Figure 1B–C). 

To verify the interaction at the predicted site, a point mutation was introduced in NsrR1 affecting the predicted helix between NsrR1 and the 5′-UTR of *all1871* (nucleotide 51, U to G). A compensatory mutation was introduced in the corresponding positions of the 5′-UTR of *all1871* (nucleotide −13 with respect to the start codon, G to C) and different combinations of the wild-type and mutated versions of both NsrR1 and 5′-UTR of *all1871* were co-expressed. Mutation of nucleotide 51 in NsrR1 reduced the interaction between NsrR1 and the 5′-UTR of *all1871*, as suggested by the lower degree of fluorescence reduction with respect to the control (Figure 1C). When the mutated version of NsrR1 was combined with the mutated version of the mRNA containing the compensatory change, base pairing was restored resulting in a stronger fluorescence reduction than with wild-type NsrR1. Because of the long region of interaction, mutation of one single nucleotide produces a small effect, but differences observed are statistically significant. These data support a direct interaction of NsrR1 with the 5′-UTR of the *all1871* mRNA that affects translation of All1871.

**Figure 2:**
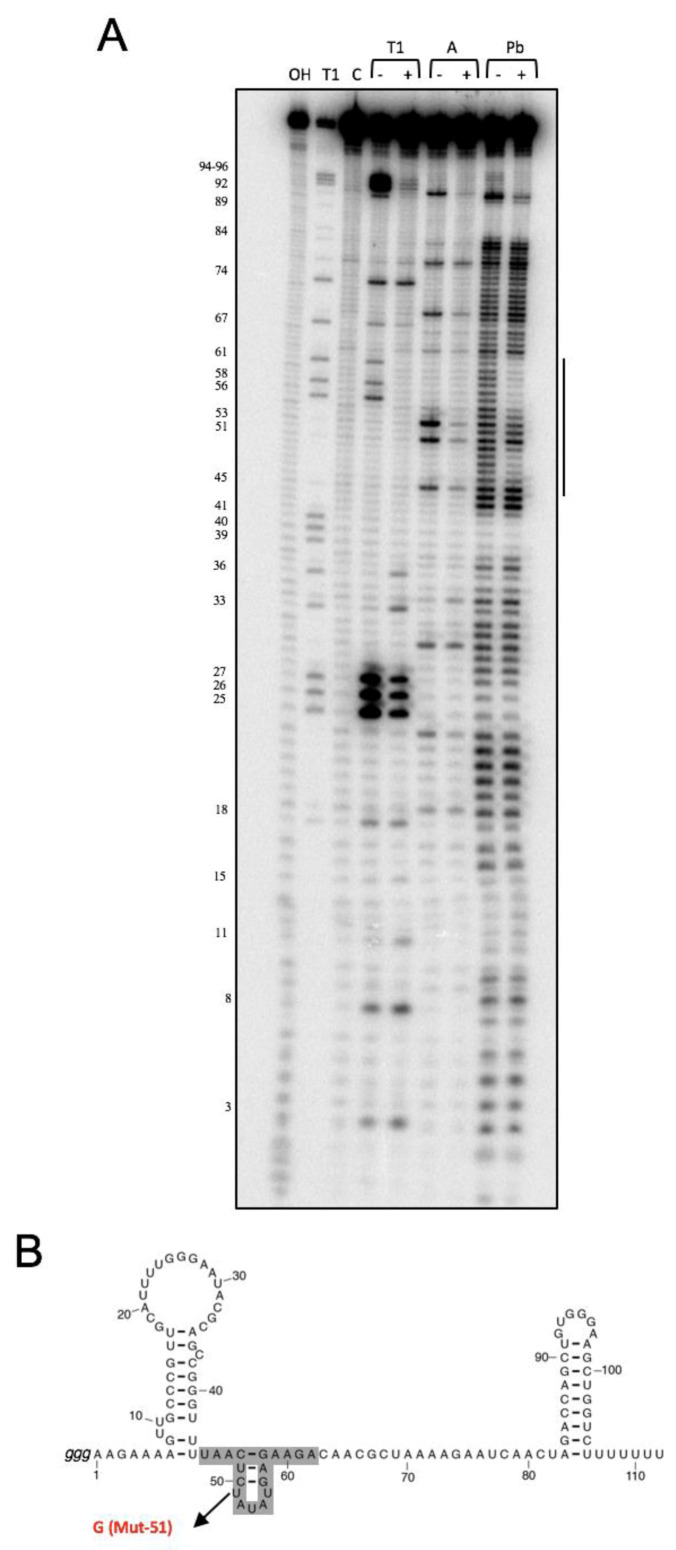
In vitro footprinting assay of the interaction between NsrR1 and *all1871* 5′-UTR. (**A**) RNase T1, RNase A or Pb(II) footprinting of the interaction between NsrR1 and the 5′-UTR of *all1871*. -/+ above lanes indicate absence/presence of *all1871* 5′ UTR. The protected area is indicated by a vertical bar. NsrR1 was 5′ end-labelled. C, untreated control; OH, alkaline ladder; T1, RNase T1 ladder. Nucleotide positions of NsrR1 are shown on the left. (**B**) The nucleotides in NsrR1 involved in the interaction with the *all1871* 5′-UTR are indicated in grey on the previously described secondary structure model of NsrR1 [10]. The nucleotide changed (U to G) in version Mut-51 of NsrR1 is also indicated.

In addition, we have studied the interaction between NsrR1 and the *all1871* mRNA by in vitro footprinting analysis. ^32^P-labelled NsrR1 was incubated with unlabeled *all1871* mRNA (a fragment extending from positions −137 to +60 with respect to the start of the coding sequence) and probed with RNase T1, RNase A or lead(II) acetate (Figure 2A). A clear footprint was detected between positions 45 and 62 of NsrR1 (highlighted in the secondary structure model, Figure 2B), in good agreement with the bioinformatic prediction (Figure 1A). These in vitro results, therefore, confirm the in vivo results obtained from the analysis in *E. coli* using the sfGFP fusion system. 

### 3.2. Expression of all1871 Is Regulated by Nitrogen Availability But Does Not Require NtcA or HetR

**Figure 3:**
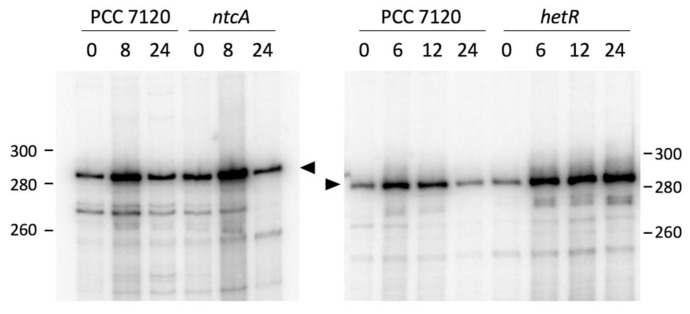
Nitrogen-regulated expression of *all1871*. Primer extension analysis of *all1871* transcripts in *Nostoc* sp. PCC 7120 and its *ntcA* mutant derivative CSE2 (left panel) or in *Nostoc* sp. PCC 7120 and its *hetR* mutant derivative DR884a (right panel). Expression was analyzed in cells grown in the presence of ammonium and transferred to medium containing no source of combined nitrogen for the number of hours indicated. Size markers (nucleotides) are indicated. Triangles point to the products corresponding to the 5′ end at position 2234072r [5].

According to global transcriptomic analyses, accumulation of the *all1871* transcript is induced in response to nitrogen deficiency [16,35]. We have analyzed transcription from the TSS identified at position 2234072r [5] in the wild type strain and in two mutant derivatives, *ntcA* mutant strain CSE2 [36] and *hetR* mutant strain DR884a [27]. Although in all strains there is significant expression in the presence of ammonium, transcription is similarly induced upon nitrogen step down in the wild type and in both mutant strains, suggesting the induction of transcription does not require NtcA or HetR (Figure 3). According to primer extension analysis, induction is transient both in the wild type and the *ntcA* strain, with a decreased amount of transcript at 24 h *vs.* 8 h after nitrogen removal, but in the *hetR* mutant the accumulation of transcript continues to be strong even at 24 h after nitrogen removal.

### 3.3. Expression of all1871 Is Reduced by NsrR1 in Nostoc sp. PCC 7120

In order to study in vivo in *Nostoc* the possible effect of NsrR1 on *all1871* expression, we used a mutant strain that lacks NsrR1 (*∆nsrR1*) [10]. Cells of strain *∆nsrR1* have no apparent difference in growth with respect to wild-type cells in media containing nitrate, ammonia, or lacking a source of combined nitrogen [10].

**Figure 4:**
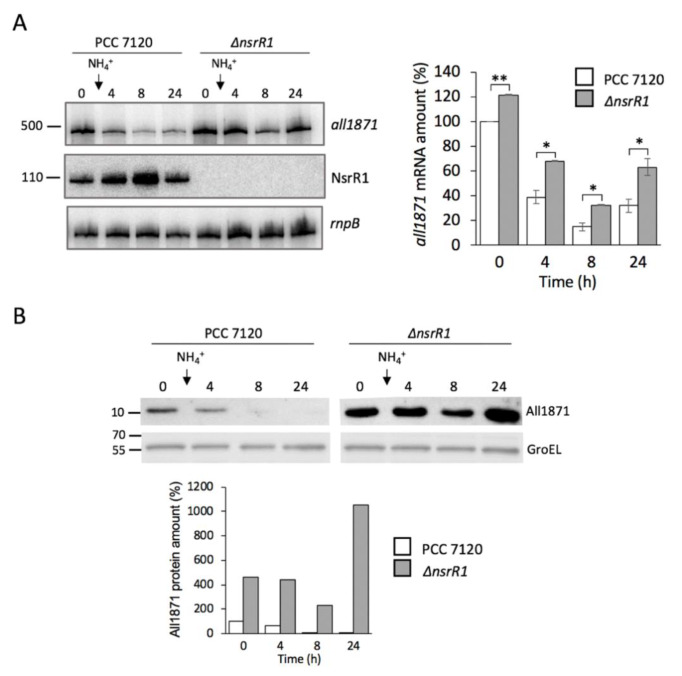
NsrR1 affects nitrogen-regulated expression of *all1871*. (**A**) Expression of *all1871* was analyzed by Northern blot in *Nostoc* sp. PCC 7120 and in a mutant strain lacking NsrR1 (∆*nsrR1*). Cells were grown in nitrogen-free liquid medium for one week (steady-state N_2_-fixing cultures) and 10 mM ammonium chloride was added to increase expression of NsrR1. Samples were taken before ammonium addition (0) and at the number of hours indicated after ammonium addition. The upper panel shows hybridization to the *all1871* probe. The middle panel shows hybridization to the probe for NsrR1. The lower panel shows hybridization to a probe for *rnpB* used as loading and transfer control. Quantification of *all1871* mRNA accumulation is indicated on the right, relative to the amount present in the wild type strain in N_2_ using the amount of *rnpB* for normalization. Results from two technical replicates were averaged. *T*-test *p*-value < 0.05*; < 0.01**. (**B**) Accumulation of the All1871 protein was determined by Western blot in samples containing 40 μg of soluble fraction from cells analyzed in (**A**). Upper panels show detection of All1871. Lower panels show detection of GroEL, used as loading and transfer control. Quantification of All1871 protein is shown at the bottom, relative to the amount present in the wild type strain in N_2_. Quantification was performed with ImageLab software (Bio-Rad) using the amount of GroEL for normalization. One representative experiment is shown.

We have previously shown that transcription of NsrR1 is only partially (and transiently) repressed upon nitrogen step down, whereas maximal expression of NsrR1 is achieved upon ammonium addition to cells growing in the absence of combined nitrogen [10]. Therefore, to maximize the difference between wild-type cells expressing NsrR1 and cells lacking NsrR1 (*∆nsrR1* strain), we chose to analyze cells grown in the absence of combined nitrogen (N_2_) and compared them with cells grown in the absence of combined nitrogen to which ammonium was added and incubation continued for 4, 8 or 24 h (Figure 4). 

As previously described, expression of NsrR1 was induced and reached its highest level 8 h after ammonium addition (Figure 4A, middle panel). Concomitantly, expression of *all1871* was repressed in the wild type, with a minimum in the sample corresponding to 8 h after ammonium addition (Figure 4A, upper panel). Furthermore, repression of *all1871* was weaker in the strain lacking NsrR1, suggesting NsrR1 has a significant negative effect on the accumulation of *all1871* mRNA in *Nostoc*, consistent with the interaction observed in the *E. coli* assay between NsrR1 and the *all1871* mRNA. The effects of NsrR1 on the accumulation of All1871 protein were also analyzed by Western blot using antibodies we have generated against purified recombinant All1871 protein. The levels of All1871 protein observed in extracts of strain *∆nsrR1* were about five-fold higher than those in the wild-type strain in the absence of combined nitrogen (Figure 4B). Consistent with the mRNA levels (Figure 4A), the reduction of the amount of All1871 protein in response to ammonium addition was stronger in the wild-type strain than in the *∆nsrR1* strain lacking NsrR1.

### 3.4. All1871 Is Differentially Expressed in Heterocysts and Required for Diazotrophic Growth But Not for Heterocyst Differentiation

**Figure 5:**
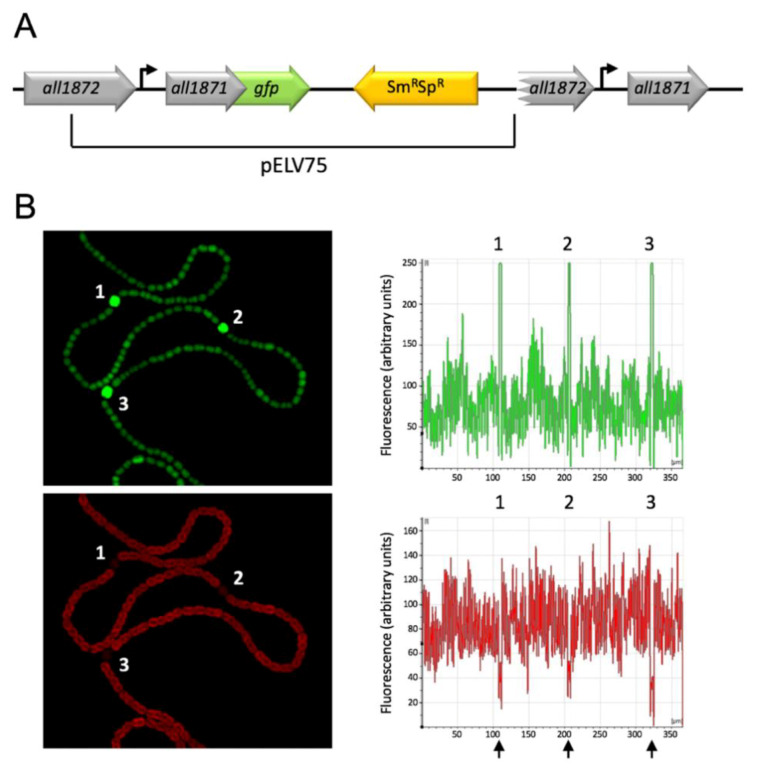
Expression of P*_all1871_*-*all1871*-*gfp* along nitrogen-fixing filaments of *Nostoc* sp. PCC 7120 bearing pELV75. (**A**) Schematic representation of plasmid pELV75 integrated by single recombination in the chromosomal region encoding *all1871*. The location of the transcriptional start site of *all1871* (bent arrows) and the segment (from -336 with respect to the start codon of *all1871*) fused to *gfp* in plasmid pELV75 are depicted. Not drawn to scale. (**B**) Confocal fluorescence image of a filament growing on top of nitrogen-free medium is shown for the green channel (upper panel, GFP fluorescence) and red channel (lower panel, autofluorescence). Quantification of the green and red signals along the filament is shown on the right of each image. Mature heterocysts are indicated with numbers. The positions of lowest autofluorescence, corresponding to heterocysts, are indicated with black arrows.

In order to obtain an insight into a possible function of All1871, we analyzed expression of *all1871* along filaments of *Nostoc*. We prepared plasmid pELV75 containing a segment from position −200 with respect to the TSS of *all1871* plus a translational fusion between the entire *all1871* gene and the *gfpmut2* gene. This plasmid was introduced in *Nostoc* by conjugation. Integration of the plasmid in the region encoding *all1871* leads to a partial duplication of this region in which the translational fusion is preceded by the natural context of *all1871* in the wild type strain (Figure 5A). 

GFP fluorescence was analyzed by confocal fluorescence microscopy of filaments growing on top of medium lacking combined nitrogen (Figure 5B). Quantification of the green signal (GPF) and the red autofluorescence along the filaments showed that although all cells showed green fluorescence, fluorescence peaks were associated with cells that had differentiated as heterocysts, as indicated by their larger size and reduced red autofluorescence. 

**Figure 6:**
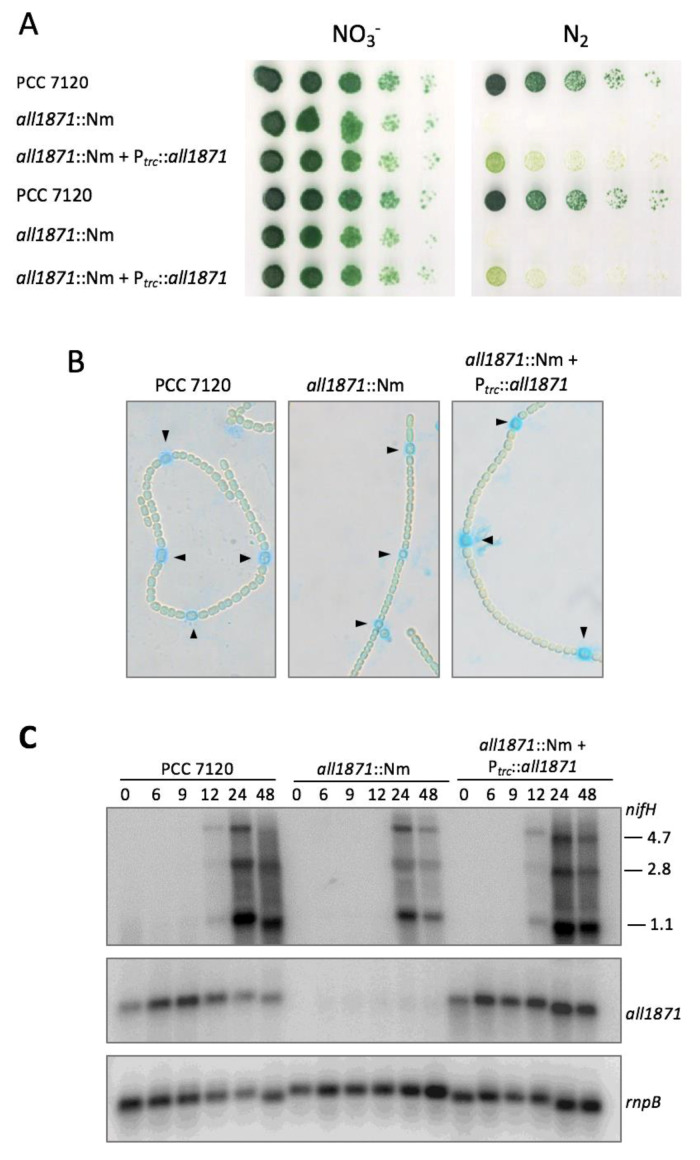
*all1871* mutants are defective in diazotrophic growth. (**A**) Cells were grown in the presence of nitrate, washed and resuspended in BG11_0_ at an OD_750_ = 0.3. Five-fold serial dilutions of liquid cultures of wild type, insertional mutant *all1871*::Nm and complemented mutant strain (*all1871*::Nm + P*_trc_*::*all1871*) were prepared and 10 µL of each dilution plated on BG11_0_ plates lacking nitrogen (N_2_) or containing nitrate (NO_3_^-^). Two different clones of each strain were analyzed. Pictures were taken after 10 days (NO_3_^-^) or 13 days (N_2_) of incubation at 30 °C. (**B**) Alcian blue staining of heterocyst polysaccharides in filaments of *Nostoc* sp. PCC 7120, insertional mutant *all1871*::Nm and complemented mutant strain (*all1871*::Nm + P*_trc_*::*all1871*), 26 h after combined nitrogen removal. (**C**) Northern blots with RNA isolated from the indicated strains at different time points (indicated in hours) after nitrogen removal and hybridized with probes for *nifH* (upper panel), *all1871* (middle panel) and *rnpB* (lower panel) as loading control. Sizes of the *nifH* transcripts are indicated on the right in kb.

We then prepared an *all1871* null mutant by interrupting the *all1871* gene with a Nm^R^ gene (Figure S2A). Complete segregation of mutant chromosomes was verified by PCR amplification and Western blot with antibodies against All1871 (Figure S2B–C). Cells of the *all1871*::Nm mutant were unable to grow on plates in the absence of combined nitrogen but showed no growth defect in the presence of combined nitrogen (nitrate) (Figure 6A). The mutation was complemented by introduction of a plasmid bearing *all1871* expressed from the *trc* promoter from *E. coli*, that provides constitutive expression in *Nostoc* (see e.g., [37]), leading to partial recovery of the wild-type phenotype, as indicated by the greenish growth of complemented cells vs. the blue-green growth of wild-type cells (Figure 6A). 

We wondered whether the observed defects in diazotrophic growth of the *all1871*::Nm strain were due to lack of heterocyst differentiation or heterocyst function. Because steady-state nitrogen-fixing cultures could not be obtained, we analyzed morphological differentiation of heterocysts 26 h after combined nitrogen removal by staining the cells with Alcian blue, a molecule that binds to the external polysaccharide layer of heterocysts. Figure 6B shows the presence of Alcian blue-stained cells in a regular pattern similar to the wild type in the mutant strain *all1871*::Nm as well as in the complemented strain *all1871*::Nm + P*_trc_*-*all1871*, indicating differentiation of heterocysts took place in both strains, at least to the relatively initial stage in which polysaccharides are deposited outside the outer membrane of the vegetative cells undergoing differentiation into heterocysts. As an indication of heterocyst maturity, transcription of the *nifHDK* genes encoding nitrogenase was also analyzed by Northern blot (Figure 6C). Transcription of *nifHDK* seems delayed and reduced in the *all1871*::Nm mutant. Again, complementation with a plasmid bearing *all1871* expressed from the *trc* promoter from *E. coli* leads to recovery of the wild-type timing of *nifHDK* expression.

Finally, we have measured the nitrogenase activity of the different strains (Table 1). Strain *all1871*::Nm had no detectable nitrogenase activity in either oxic or anoxic conditions. This result excludes the possibility that the absence of activity in strain *all1871*::Nm was due to a defect in the maturation of heterocyst envelopes that results in the presence of inactivating O_2_ amounts inside the heterocyst.

## 4. Discussion

Small RNAs are important components in regulatory networks that involve classical transcription factors. For instance, in *E. coli*, members of the CRP/FNR family of transcriptional regulators are known to control the expression of several small RNAs, all of them contributing to the regulatory effects exerted by these two major transcription factors [2]. Cyanobacterial transcription factors also regulate the expression of small RNAs that exert regulatory functions involved in the adaptation to different environmental situations [38]. For instance, the transcriptional regulator RpaB and small RNA PsrR1 constitute a feed-forward loop controlling acclimation to different light intensities [39]. Accumulating evidence concerning nitrogen-regulated non-coding RNAs indicates this type of molecules is involved in NtcA-mediated post-transcriptional regulation [5,9]. One case analyzed in detail is NsiR4, whose transcription is induced in response to nitrogen deficiency and is involved in post-transcriptional regulation of glutamine synthetase [8]. Expression of another sRNA, NsrR1, is repressed by NtcA in response to nitrogen deficiency. Among predicted targets of NsrR1 are *nblA* [10], and *all1871*, whose interaction with NsrR1 we describe in this work. 

Interaction between homologs of NsrR1 and homologs of *all1871* is conserved in many cyanobacteria encoding NsrR1 (Figure S1), consistent with the observation that *all1871* has the highest score in CopraRNA predictions of candidates to be regulated by NsrR1 [10]. Using an in vivo reporter system established in *E. coli* we demonstrate that NsrR1 represses translation, leading to reduced expression of an *all1871*::sfGFP fusion in the presence of NsrR1. A direct interaction of NsrR1 with the predicted region in the 5′-UTR of *all1871* was also verified by a point mutation in the region involved (Figure 1) and by in vitro footprinting experiments (Figure 2).

We have demonstrated higher accumulation of *all1871* transcripts in a strain of *Nostoc* sp. PCC 7120 that lacks NsrR1 (∆*nsrR1*) than in wild-type cells cultured in the absence of combined nitrogen (Figure 4A). Upon addition of ammonium, that induces NsrR1 expression, the amount of *all1871* mRNA is strongly reduced in the wild type but not in the ∆*nsrR1* strain. Similarly, the amount of All1871 protein, detected with a specific antibody (Figure 4B), is much higher in cells lacking NsrR1 than in the wild-type strain. Upon the addition of ammonium, the amount of All1871 protein is reduced in the wild type, becoming barely detectable 8 h after ammonium addition, but only slightly reduced in the ∆*nsrR1* strain. The magnitude of the repression upon ammonium addition is higher at the protein level than at the transcript level, in agreement with NsrR1 inhibiting translation through its interaction with the 5′-UTR of *all1871*. The reduced accumulation of *all1871* mRNA in the presence of NsrR1 can be attributed to destabilization of the mRNA when translation is inhibited.

**Figure 7:**
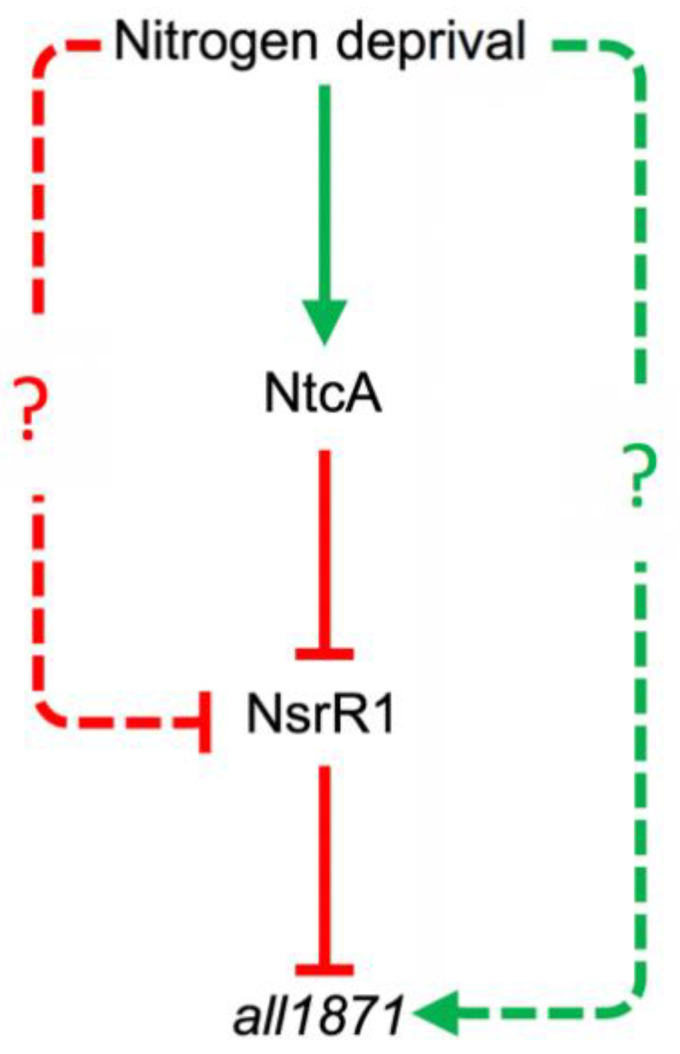
Schematic representation of elements involved in the regulation of expression of *all1871*. Green arrows represent positive effects. Red arrows represent negative effects. Dashed lines represent mechanisms operated by currently unknown factors (question marks).

Upregulation of *all1871* upon nitrogen deprivation would be mediated, at least in part, by alleviating the repression exerted by NsrR1, whose transcription is repressed upon nitrogen stress. NsrR1 repression is only partially dependent on NtcA, with an additional factor possibly involved [10], explaining that up-regulation of *all1871* is independent of NtcA (Figure 3). The observation that there is partial repression of *all1871* expression upon ammonium addition even in the *∆nsrR1* mutant strain (Figure 4) points to some additional factor(s) being involved in regulation of *all1871*. In any case, because the interaction between NsrR1 and *all1871* takes place at the translation initiation region, even if another regulatory mechanism influences transcription of *all1871*, the accumulation of the All1871 protein would be ultimately regulated by nitrogen availability through NsrR1 (Figure 7).

By means of a translational fusion to the *gfp* gene we have shown higher expression of *all1871* in heterocysts than in vegetative cells (Figure 5). We have also shown that expression of *all1871* does not require HetR (Figure 3) suggesting it is not heterocyst-specific. Taken together these observations point to the operation of post-transcriptional regulatory mechanisms leading to differential accumulation of *all1871* transcripts in heterocysts *vs.* vegetative cells. Because NtcA is subjected to differential accumulation in heterocysts [31,40], the amount of NsrR1 is also expected to be different. A higher amount of NtcA in heterocysts would result in lower levels of NsrR1, and consequently increased translation of All1871 in these specialized cells. NsrR1 would thus contribute to differential expression in heterocysts of a transcript found to be required for diazotrophic growth. Whether the stronger accumulation of All1871-GFP protein in heterocysts (Figure 5) is only a consequence of reduced post-transcriptional regulation by NsrR1 or involves additional differential regulation in these specialized cells, is currently unknown.

All1871 is conserved in many unicellular and filamentous strains of cyanobacteria. Its function is unknown and it does not contain domains of known function. Strong accumulation in heterocysts together with the observation that its expression is regulated by NsrR1, a nitrogen-repressed sRNA, suggest a possible role in the response to nitrogen stress and/or in heterocyst function. A mutant lacking All1871 (*all1871*::Nm) could not grow in media lacking combined nitrogen (Figure 6A). The inability to grow in media lacking combined nitrogen of the *all1871*::Nm strain is only partially complemented by constitutive expression of *all1871* from P*_trc_* (Figure 6A). Such phenotype could be explained by toxicity due to unregulated expression of *all1871* in this strain, in contrast to the transient induction observed in the wild type upon nitrogen step-down. It can also be speculated that constitutive expression of *all1871* in the complemented strain could lead to sequestration of NsrR1 affecting accumulation of other mRNAs also regulated by NsrR1, such as *nblA* [10]. However, it has been previously shown that cells lacking NsrR1 have no apparent phenotypic differences with respect to wild-type cells [10]. Despite its ability to develop Alcian blue-stained cells (Figure 6B), suggestive of heterocyst differentiation, and the induction of nitrogenase transcription (Figure 6C), *all1871*::Nm filaments lacked nitrogenase activity (Table 1), indicating that All1871 is required for heterocyst function through currently unknown mechanisms. We have observed that in the absence of combined nitrogen, filaments of the *all1871*::Nm strain are extensively fragmented in liquid media (not shown). Because exchange of metabolites between vegetative cells and heterocysts is a requisite for sustained nitrogen fixation, filament fragmentation would preclude diazotrophic growth of the *all1871*::Nm strain.

In summary, in this work we describe a new gene required for diazotrophic growth, although not for heterocyst differentiation, and characterize its regulation by NsrR1, a nitrogen-regulated small RNA involved in acclimation to nitrogen deficiency. 

## Figures and Tables

**Table 1:** Nitrogenase activity of different *Nostoc* strains.

Strain	Nitrogenase Activity ^1^ (µmol Ethylene·h^−^^1^·mg Chl^−^^1^)	
	Oxic Conditions	Anoxic Conditions
*Nostoc* sp. PCC 7120	10.50 ± 3.49	10.64 ± 2.05
*all1871*::Nm	0.00 ± 0.00	0.00 ± 0.00
*all1871*::Nm + P*_trc_*::*all1871*	3.66 ± 1.67	5.05 ± 3.41

^1^ Nitrogenase activity was measured in cultures grown with nitrate and transferred for 24 h to nitrogen-free medium. Data are the average and standard deviation of assays performed with two independent cultures of *Nostoc* sp. PCC 7120 and strain *all1871*::Nm + P*_trc_*::*all1871* or eight independent cultures of strain *all1871*::Nm.

## Supplementary Materials

The following are available online at https://www.mdpi.com/2075-1729/10/5/54/s1: Figure S1: Conservation in cyanobacteria of the predicted interaction between the mRNA of *all1871* and NsrR1; Figure S2: Construction of mutant *all1871*::Nm; Table S1: Strains; Table S2: Oligonucleotides; Table S3: Plasmids; Table S4: Sequences of inserts in plasmids containing NsrR1 used for verification in *E. coli*; Table S5: Sequences of inserts in the *all1871*-sf*gfp* fusion plasmids; Table S6: Sequences of templates used for in vitro transcription.
