# Supplementary material for: The Nitrogen Stress-Repressed sRNA NsrR1 Regulates Expression of all1871, a Gene Required for Diazotrophic Growth in Nostoc sp. PCC 7120
Source: Life (Basel). 2020 Apr 29;10(5):54. doi: 10.3390/life10050054 (PMC7281752; doi:10.3390/life10050054)
Supplement: Supplementary file 1 [file life-10-00054-s001.pdf]

life

Supplemental Material for The Nitrogen Stress-Repressed sRNA NsrR1 Regulates Expression of *all1871*, a Gene Required for Diazotrophic Growth in *Nostoc* sp. PCC 7120

Isidro Álvarez-Escribano, Manuel Brenes-Álvarez, Elvira Olmedo-Verd, Agustín Vioque and Alicia M. Muro-Pastor*

Instituto de Bioquímica Vegetal y Fotosíntesis, Consejo Superior de Investigaciones Científicas and Universidad de Sevilla, 41092 Sevilla, Spain; isidroae9@hotmail.com (I.A-E.); mabreal92@gmail.com (M.B-A.); eolmedoverd@hotmail.com (E.O-V.); vioque@us.es (A.V.)

* Correspondence: alicia@ibvf.csic.es ; Tel.: +34-954489521

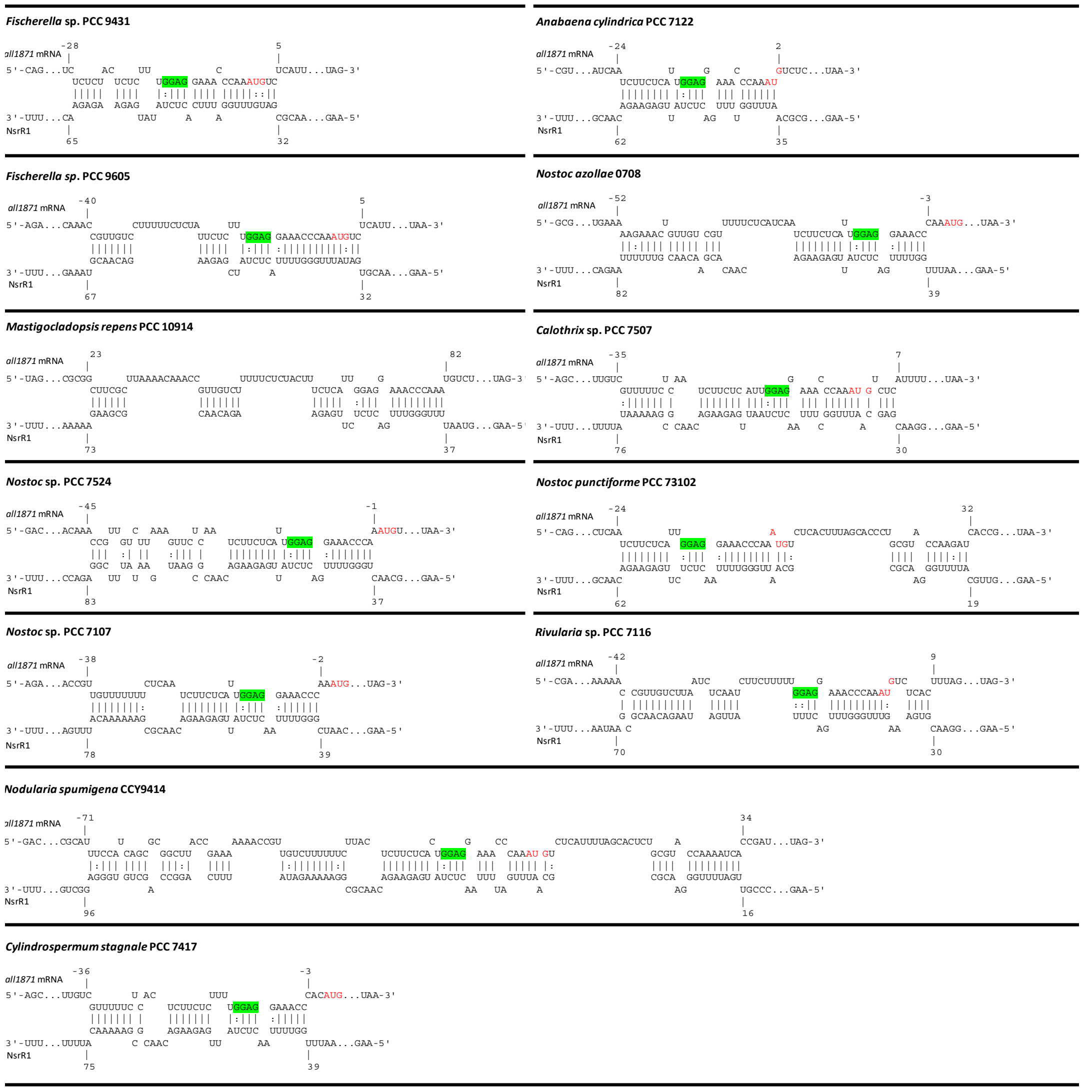

Figure S1. Conservation in cyanobacteria of the predicted interaction between the mRNA of *all1871* and NsrR1. Potential interaction between NsrR1 homologs and mRNAs corresponding to *all1871* homologs (analyzed from 200 nucleotides upstream of the start codon to the stop codon of the coding sequence) was computed for 22 cyanobacteria that contain NsrR1 [10] using IntaRNA software [34]. Nucleotide positions in the mRNAs are numbered from first nucleotide of the coding sequence, negative upstream to positive downstream. AUG start codons (red) and putative Shine–Dalgarno sequences (green shading) are indicated. Only those strains with a predicted stable interaction are shown.

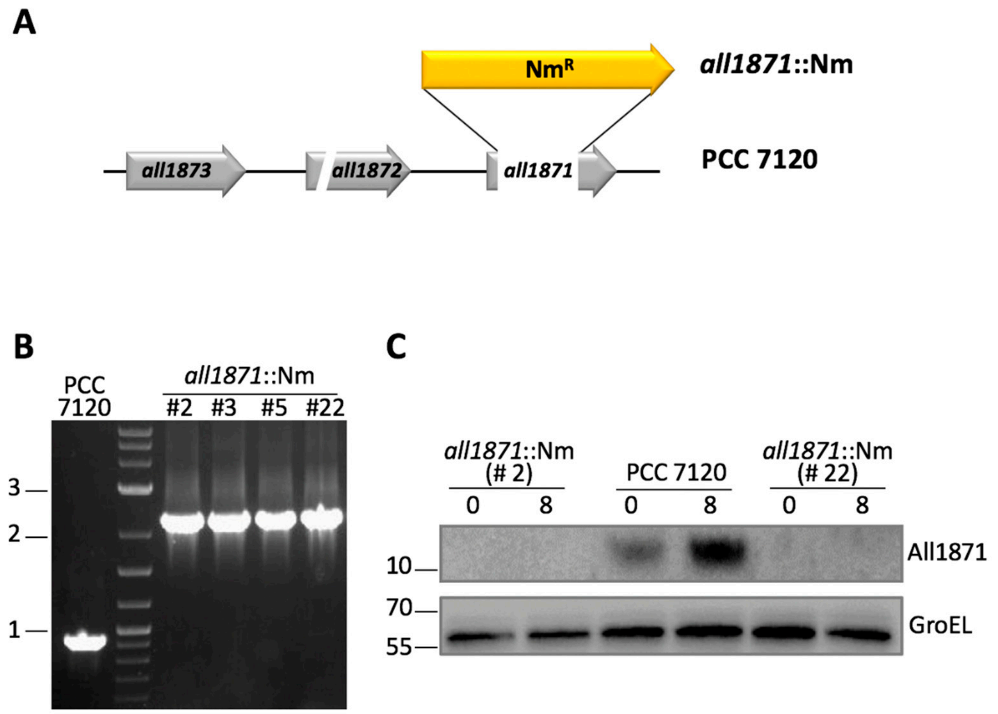

Figure S2. Construction of mutant *all1871*::Nm. (**A**) Schematic representation of the replacement of the central portion of *all1871* by Nm^R gene. (**B**) Verification of segregation of the mutation introduced. Polymerase chain reaction (PCR) amplification of the *all1871* region in the wild-type strain (expected size: 0.9 kb) and four independent isolates of the *all1871*::Nm strain (expected size: 2.2 kb) was carried out using oligonucleotides 162 and 180 (Table S2). (**C**) Accumulation of the All1871 protein was determined by Western blot in samples containing 40 mg of soluble fraction from wild type and two isolates of the *all1871*::Nm strain. Upper panel shows detection of All1871. The lower panel shows detection of GroEL, used as loading and transfer control.

Table S1. Strains.

Name	Description	Reference
Escherichia coli		
DH5α	Used for routine transformation	Hanahan, 1983[a]
BL21(DE3)-RIL	Cm^R, used for overexpression of recombinant proteins	Agilent Technologies
Nostoc sp.		
PCC 7120	Wild type	Pasteur Culture Collection
CSE2	*ntcA* null mutant (*ntcA*::C.S3)	[36]
DR884a	*hetR* null mutant (*hetR*::*luxAB*)	[27]
Δ*nsrR1*	*nsrR1* gene deleted	[10]
all1871::Nm	*all1871* mutant	This work
all1871::Nm + P_{trc}-*all1871*	*all1871* gene under control of the *trc* promoter in *all1871*::Nm background	This work

[a] Hanahan, D. Studies on transformation of *Escherichia coli* with plasmids. *J. Mol. Biol.* **1983**, *166*, 557-580.

Table S2. Oligonucleotides.

Name	Sequence (5'–3')	Used for
158	CATCTGCCTCTGCCTCTTCTG	PCR to generate radioactive probe of NsrR1
159	CCTTCCTTGTAGGCAGTCGAG	
161	GTCCAAAACTGCAACGATGTC	Primer extension of *all1871* transcripts
162	GGATCCGATGGTGGTACATCCAG	PCR of the region *all1871* for mutant construction in *Nostoc*
163	TTGATCAACTCGAGGATTTCGGCATCGGTG	
164	GAAATCCTCGAGTTGATCAACTCCATCAACC	
165	GGATCCAGTTTACCCATATCG	
180	CAGCCAAGGTTTTGTTAACAG	Verification of *all1871* mutants in *Nostoc*
189 (PLlacOB)	CGCACTGACCGAATTCATTAA	Plasmid backbone amplification from pZE12-luc
190 (PLlacOD)	GTGCTCAGTATCTTGTTATCCG	
197	5'P-AAGAAAAGTTGCCCGTTGC	PCR of NsrR1 for cloning in pZE12-luc
198	GTTTTTTCTAGACAGAGACACCACGTAAAACGACT	
247	GTTTTATGCATTCCCTGCTAACCCAGGGG	PCR of 5'-UTR of *all1871* for cloning in pXG10-SF, used also as probe
248	GTTTTTCTAGAGGAAGCTTTGAGGATTTCGGC	
295	TTTAACTCGATATGAGAAGACAACGC	Mutagenesis of NsrR1 to generate Mut-51
296	TCATATCGAGTTAAAACCCGGCTGCG	
303	TTCTCATTCGAGGAAACCCAAATGTC	Mutagenesis of *all1871* to generate Comp-51
304	TTCCTCGAATGAGAAGATTGAGAAAAAG	
335	GTTTTCCATGGCTCACTTTAGCACTCTCCGC	PCR of *all1871* for cloning in pET28a
336	GTTTTCTCGAGTTGCAATACCAACTTCACATTG	
569	GTTTTGAATTC**TAATACGACTCACTATA**GGGAAGA AAAGTTGCCCGTTGC	PCR of template for in vitro transcription of NsrR1
570	GTTTTGGATCCTTTAAAAAAAGACCAGCTTCCCAC	
597	GAATTC**TAATACGACTCACTATA**GGGTCCCTGCTA ACCCAGGGG	PCR of template for in vitro transcription of *all1871* 5'-UTR
598	GGAAGCTTTGAGGATTTCGG	
451	GTTTTATCGATAGTTGCGGCAATGTACGAAGC	Construction of *all1871-gfpmut2* translational fusion
596	GTTTGATATCTTGCAATACCAACTTCACATTGGC	
735	GTTTTCTCGAGTCATTGCACCAGATGGTTAC	Construction of plasmid bearing *all1871* under the *trc* promoter
787	GTACTGCAAGGGGCGTGGCT	PCR to generate radioactive probe of *nifH*
788	CCTATTGGTAGCTTCTGCGGG	

Sequences are given in 5'→3' direction; 5'P denotes a 5' monophosphate. Underlined, restriction sites used for cloning. In red nucleotide changes with respect to the native wild type sequence. The T7 promoter sequence in oligonucleotides 569 and 597 is in bold.

Table S3. Plasmids.

Name	Description	Reference
pET28a(+)	Km^R, vector for his-tagged protein expression	Novagen
pSpark	Ap^R, vector for cloning PCR products	Canvax Biotech
pAVN1	Ap^R, PCR fragment generated with primers 197 and 198, containing *nsrR1*, digested with XbaI and cloned in the vector backbone generated by PCR of pZE12-luc with primers 189 (PLlacOB) and 190 (PLlacOD)	[10]
pCSEL21	Ap^R, plasmid containing *gfpmut2* gene, to construct translational fusions	[31]
pCSRO	Sm^RSp^R, *sacB*-containing vector for conjugation of *Nostoc*	[26]

pCSV3	Sm^{R}Sp^R, mobilizable vector for conjugation of *Nostoc*	[31]
pJV300	Ap^R, derivative of pZE12-luc for transcription of a control, 50-nt RNA	[20]
pMBA37	Ap^{R}Sm^{R}Sp^R, containing *trc* promoter of *E. coli*	[15]
pRL278	NmR, *sacB*-containing vector for conjugation of *Nostoc*	[27]
pXG0	Cm^R, control plasmid without GFP	[18]
pXG10-SF	Cm^R, vector for the generation of sfGFP fusions	[19]
pZE12-luc	Ap^R, plasmid for cloning sRNAs	Lutz *et al.*, 1997 [a]
pELV73	Ap^R, derivative of pCSAM147, containing a fragment amplified with oligonucleotides 451 and 596, that includes from position -200 with respect to TSS to the stop codon of *all1871* substituted with a EcoRV site, cloned in phase with the GFPmut2 protein.	This work
pELV75	Sm^{R}Sp^R, pCSV3 derivative containing, in the unique EcoRI site, the *all1871-gfpmut2* translational fusion in pELV73	This work
pIAE9	Cm^R, PCR fragment generated with primers 247 and 248, containing *all1871* 5′-UTR+60 bp, digested with NsiI and XbaI, and cloned in pXG10-SF digested with NsiI and NheI	This work
pIAE20	Ap^R, same as pAVN1 but with a U to G change at position 51 of NsrR1 (Mut-51)	This work
pIAE22	Cm^R, same as pIAE9 but with a G to C change at position -13 of the 5'-UTR (Comp-51)	This work
pIAE30	Km^R, PCR fragment generated with primers 335 and 336, containing the *all1871* gene from *Nostoc*, digested with NcoI and XhoI and cloned in pET28a(+) digested with the same enzymes	This work
pIAE65	Ap^{R}Sm^{R}Sp^R, derivative of pMBA37 with *all1871* expressed from the *trc* promoter of *E. coli*	This work
pSAM147	Ap^R, derivative of pCSEL21	[30]
pSAM318	Ap^R, pSpark derivative containing a fragment of the *all1871* locus with the central portion of *all1871* deleted	This work
pSAM324	Sm^{R}Sp^R, pCSRO derivative containing a BamHI fragment from pSAM318 corresponding to the *all1871* region with the *all1871* gene deleted	This work
pSAM326	Sm^{R}Sp^R Nm^R, derivative of pSAM324 with Nm^R gene inserted in the *all1871* locus	This work

[a] Lutz, R.; Bujard, H. Independent and tight regulation of transcriptional units in *Escherichia coli* via the LacR/O, the TetR/O and AraC/I1-I2 regulatory elements. *Nucleic Acids Res.* **1997**, *25*, 1203-1210.

Table S4. Sequences of inserts in plasmids containing NsrR1 used for verification in *E. coli*.

Plasmid	Sequence	Description
pAVN1	AAGAAAAGTTGCCCGTTGCATTTTGGGAATACGCAGCCGGGTTTTA ACTCTATATGAGAAGACAACGCTAAAAGAATCAACTAGACCAGCT GTGGGAAGCTGGTCTTTTTTTCCGTACATACACGTTTGACCAATAGT CGTTTTACGTGGTGTCTCTGTCTAGA	NsrR1 WT
pIAE20	AAGAAAAGTTGCCCGTTGCATTTTGGGAATACGCAGCCGGGTTTTA ACTCGATATGAGAAGAGAACGCTAAAAGAATCAACTAGACCAGCT GTGGGAAGCTGGTCTTTTTTTCCGTACATACACGTTTGACCAATAGT CGTTTTACGTGGTGTCTCTGTCTAGA	NsrR1 Mut-51

Grey shadowed letters indicate the *nsrR1* sequence. Mutation introduced is marked in red. XbaI restriction site used for cloning is highlighted in blue.

Table S5. Sequences of inserts in the *all1871*-sf*gfp* fusion plasmids.

Plasmid	Sequence	Description
pIAE9	atgcaTTCCCTGCTAACCCAGGGGGAAAGGCTAGCCAACAAAAGCT AGCAGTTACGAGAAAAAAGCCGCTCCCACATTATGCGCGGCTTCC CTGAAAACAAACGTTGTCCTTTTTCTCAATCTTCTCATTGGAGGAA ACCCAAATGTCTCACTTTAGCACTCTCCGCACCAAAATCACCGAT GCCGAAATCCTCAAAGCTTCCTctagc	*all1871* WT
pIAE22	atgcaTTCCCTGCTAACCCAGGGGGAAAGGCTAGCCAACAAAAGCT AGCAGTTACGAGAAAAAAGCCGCTCCCACATTATGCGCGGCTTCC CTGAAAACAAACGTTGTCCTTTTTCTCAATCTTCTCATTCGAGGAA ACCCAAATGTCTCACTTTAGCACTCTCCGCACCAAAATCACCGAT GCCGAAATCCTCAAAGCTTCCTctagc	*all1871* Comp-51

Nostoc gene sequences are capitalized, in which black letters correspond to 5′UTR parts and green letters to ORF parts, respectively. NsiI and NheI/XbaI sites that were used for cloning are highlighted in blue and magenta, respectively. The TSS is highlighted in red and the start codon is underlined. The nucleotide changed with respect to the native wild-type sequence is in red.

Table S6. Sequences of templates used for in vitro transcription.

Template	Sequence
all1871 WT	**GGG**TCCCTGCTAACCCAGGGGGAAAGGCTAGCCAACAAAAGCTAGCAGTTAC GAGAAAAAAGCCGCTCCCACATTATGCGCGGCTTCCCTGAAAACAAACGTTGT CCTTTTTCTCAATCTTCTCATTGGAGGAAACCCAAATGTCTCACTTTAGCACTCT CCGCACCAAAATCACCGATGCCGAAATCCTCAAAGCTTCC
NsrR1 WT	**GGG**AAGAAAAGTTGCCCGTTGCATTTTGGGAATACGCAGCCGGGTTTTAACTCT ATATGAGAAGACAACGCTAAAAGAATCAACTAGACCAGCTGTGGGAAGCTGGT CTTTTTTT

In bold, non-encoded Gs added for efficient in vitro transcription. The initiation codon of *all1871* is underlined.

© 2020 by the author. Licensee MDPI, Basel, Switzerland. This article is an open access article distributed under the terms and conditions of the Creative Commons Attribution (CC BY) license (http://creativecommons.org/licenses/by/4.0/).
